# Concomitant leukocytosis and lymphopenia predict significant pathology at CT of acute abdomen: a case-control study

**DOI:** 10.1186/s12873-019-0227-4

**Published:** 2019-01-18

**Authors:** Alexandra Platon, Chloe Frund, Laura Meijers, Thomas Perneger, Elisabeth Andereggen, Minerva Becker, Alice Halfon Poletti, Olivier T. Rutschmann, Pierre-Alexandre Poletti

**Affiliations:** 1 Department of Radiology, University Hospital of Geneva, 4 rue Gabrielle-Perret-Gentil, 1205 Geneva, Switzerland; 2 Division of Clinical Epidemiology, University Hospital of Geneva, 4 rue Gabrielle-Perret-Gentil, 1205 Geneva, Switzerland; 3 Department of Community, Primary Care and Emergency Medicine, University Hospital of Geneva, 4 rue Gabrielle-Perret-Gentil, 1205 Geneva, Switzerland

**Keywords:** Acute abdominal pain, Computed tomography, Emergency, Leukocytosis, Relative lymphopenia

## Abstract

**Background:**

Acute abdominal pain accounts for about 10% of emergency department visits and has progressively become the primary indication for CT scanning in most centers. The goal of our study is to identify biological or clinical variables able to predict or rule out significant pathology (conditions requiring urgent medical or surgical treatment) on abdominal CT in patients presenting to an emergency department with acute abdominal pain.

**Methods:**

This was a retrospective cohort study performed in the emergency department of an academic center with an annual census of 60′000 patients. One hundred and-nine consecutive patients presenting with an acute non-traumatic abdominal pain, not suspected of appendicitis or renal colic, during the first semester of 2013, who underwent an abdominal CT were included.

Two medical students, completing their last year of medical school, extracted the data from patients’ electronic health record. Ambiguities in the formulations of clinical symptoms and signs in the patients’ records were solved by consulting a board certified emergency physician. Nine clinical and biological variables were extracted: shock index, peritonism, abnormal bowel sounds, fever (> 38 °C), intensity and duration of the pain, leukocytosis (white blood cell count >11G/L), relative lymphopenia (< 15% of total leukocytes), and C-reactive Protein (CRP). These variables were compared to the CT results (reference standard) to determine their ability to predict a significant pathology.

**Results:**

Significant pathology was detected on CT in 71 (65%) patients. Only leukocytosis (odds ratio 3.3, *p* = 0.008) and relative lymphopenia (odds ratio 3.8, *p* = 0.002) were associated with significant pathology on CT. The joint presence of these two anomalies was strongly associated with significant pathology on CT (odds ratio 8.2, *p* = 0.033). Leukocytosis with relative lymphopenia had a specificity of 89% (33/37) and sensitivity of 48% (33/69) for the detection of significant pathology on CT.

**Conclusion:**

The high specificity of the association between leukocytosis and relative lymphopenia amongst the study population suggests that these parameters would be sufficient to justify an emergency CT. However, none of the parameters could be used to rule out a significant pathology.

## Background

Acute abdominal pain accounts for about 10% of emergency department visits [1] and has progressively become the primary indication for CT scanning in most centers [2], due to the diagnostic accuracy of CT in most cases of acute abdominal pain [3, 4]. It has been previously shown that CT imaging results in a change in the management plan of more than 40% of cases when compared with clinical evaluation prior to CT [3].

However, the liberal use of CT in emergency departments raises the issue of potential overuse in acute abdominal pain cases, as it exposes patients to the risks associated with ionizing radiation, along with the injection of contrast medium [5]. Furthermore, a prospective, randomized study revealed that routine CT scanning for every patient presenting to the emergency department with abdominal pain is not cost effective [6] .

Though there are well-established guidelines regarding the indications for CT in cases of suspected cholecystitis [7], appendicitis [8] and renal colic [9], based on medical history, clinical and laboratory tests and, in some instances, ultrasound, the same cannot be said for other causes of acute abdominal pain [4, 10]. Acute abdominal pain can have multiple etiologies, from the most benign (functional pains, constipation) to the most severe (bowel obstruction, hollow organ perforation, mesenteric ischemia, etc.), between which it is unfortunately not always easy to differentiate solely on the basis of medical history and clinical examination at admission [10–16]. The triage of patients with acute abdominal pain to CT is usually a long process, which risks delaying treatment and slowing patient flow in already overcrowded emergency department.

This study sought to analyze whether an association between clinical and biological variables, among examinations routinely performed on patients presenting with not well defined acute abdominal pain, could predict or rule out the presence of a significant intra-abdominal condition on CT, namely one requiring urgent medical or surgical treatment.

## Methods

### Study design and settings

This retrospective study was approved by the institutional review board of our hospital (CER-14-020). As the study was based on a review of medical records, informed written consent was not required.

The study was undertaken in a university emergency department with an annual volume of around 61,000 adult patients (> 16 years), of which 8% (4900) presenting with abdominal pain.

### Patient selection

For patients presenting with acute abdominal pain, the decision to perform a radiological examination is taken jointly by the resident physician and her/his supervisor, based on patient medical history, clinical examination and laboratory test results.

Our study included adult patients (> 16 year-old), presenting with not well defined non-traumatic acute abdominal pain for whom an intravenous contrast-enhanced CT scan was ordered as initial imaging examination at admission. Patients presenting with suspected renal colic, appendicitis, or cholecystitis were excluded from the study, given that they benefit from a standardized management, with routine low-dose CT scanning without injection of iodinated contrast medium for suspected renal colic or appendicitis [17, 18], or abdominal ultrasound for suspected cholecystitis [7].

### Data collection

The study population was identified by two medical students (CF, LM), completing their last year of medical school, under supervision of a board certified radiologist, by electronic search of the radiology department database, using the keywords “acute abdominal pain”, among all patients referred for CT in the emergency department across a period of 20 weeks, which corresponds to the rotation duration of a single team of emergency physicians in our center. During this period, a total of 2041 patients presented to the emergency department for acute abdominal pain. The electronic search identified 971 CT reports. The following exclusion criteria were applied: patients for whom CT was ordered for suspected appendicitis (*n* = 315), for detection of stones in the biliary tract after ultrasound (*n* = 3), for suspected renal colic (*n* = 332) and for trauma (*n* = 212).

A total of 109 consecutive patients met our inclusion criteria and formed the study population.

Nine clinical, hematological and biological parameters systematically tested at patients’ admission were extracted from patients’ files and transcribed in a dichotomous form (normal or abnormal). This extraction was performed by the same medical students who already identified the study population. Ambiguities in the formulations of clinical symptoms and signs in the patients’ records were solved by consulting a board certified emergency physician (OTR). The extracted parameters are listed below (the thresholds for abnormality are indicated in parenthesis):

1) shock index (heart rate/systolic blood pressure > 0.7), 2) fever (≥38 °C), 3) intense pain (visual analogue scale ≥7/10), 4) long-lasting pain (> 6 h), 5) peritonism (abdominal guarding and/or tenderness to palpation), 6) abnormal bowel sounds (metallic sounds, reduced/absence of sounds), 7) leukocytosis (leukocyte count > 11 G/L), 8) relative lymphopenia (lymphocyte count < 15% of total leukocytes), and 9) elevated C-reactive protein (CRP) (> 11 mg/l).

### CT technique

CT examinations were performed on a 64-row equipment (Discovery CT 750 HD, General Electric Company, Milwaukee, WI, USA) from lung base to pelvis. Patients received an intravenous bolus of 120 mL non-ionic contrast material (Iohexol, 300 mg I/mL, GE Healthcare AG, Opfikon, Switzerland), at a flow rate of 3.5 mL/second using a power injector, with a delay of 60 s before initiating CT data acquisition, followed by a 30 mL saline flush at the same flow rate.

The following CT acquisition parameters were used: 64 × 1.25 mm collimation, 0.9 pitch, 0.7-s gantry rotation period, 120 kV tube potential, automated tube current modulation, 2-1 mm reconstruction thickness, 40% adaptive statistical iterative reconstruction (ASIR).

### Reference standard

The diagnosis on the final CT report, validated by the attending emergency radiologist on call, was considered reference standard. The CT diagnosis, in the frame of this study, was considered positive in the presence of a significant pathology. A significant pathology was defined prior to the beginning of the data collection in consensus by the attending physicians representing the units of emergency medicine (AHP, OTR), emergency surgery (EA) and emergency radiology (AP, PAP), as a condition which explained the abdominal pain and required medical or surgical treatment. A CT without pathological finding, or which showed a condition already known before admission and unrelated to the acute abdominal pain, was considered negative, as well as CT showing only signs of constipation.

### Statistical analysis

The data were recorded and analyzed using a dedicated statistical software pack (IBM®SPSS® Statistics 22, IBM Corporation, USA). First, descriptive statistics were generated. Continuous values were expressed as mean ± standard deviation. Associations of significant findings on CT at admission and clinical and biological variables were performed using the Chi-squared test for categorical variables and a T-test for the means. Logistic regression analysis was performed to identify the variables associated with an increased likelihood of significant findings on CT examinations. The level of statistical significance for all tests was defined as a *p* value < 0.05.

## Results

Our study population consisted of 109 patients, 60 women and 49 men, with a mean age of 59 years (range 16–94, inter-quartile range (IQR) 46–78). There were 71 (65%) positive CT examinations. The group of patients with a positive CT was made up of 38 (54%) women and 33 (46%) men, with a mean age of 57 (range 16–93, IQR 41–77). The group of patients with a negative CT was made up of 22 (58%) women and 16 (42%) men, with a mean age of 62 years (range 20–94, IQR 50–79).

The most commonly found pathologies on CT in the study population were: ileitis/colitis (of infectious, inflammatory or ischemic causes) (*n* = 25), small bowel obstruction (*n* = 10), pancreatitis (*n* = 6), and diverticulitis (*n* = 5). The distribution of diagnoses for the positive CT scans is recorded in Table 1.One case of appendicitis, one case of renal colic, 4 cases of pyelonephritis and 6 cases of pancreatitis were identified on CT; these diagnoses had not been suspected before the CT scan was ordered.

**Table 1:** Significant diagnoses on abdominal computed tomography

CT diagnoses	71 N (%)
Colitis, enteritis, inflammatory bowel disease	25 (35)
Small bowel occlusion	10 (14)
Pancreatitis	6 (9)
Diverticulitis	5 (7)
Hollow viscus perforation	4 (6)
Digestive tumors	4 (6)
Biliary tract diseases (cholangitis, choledocal obstruction)	4 (6)
Pyelonephritis	4 (6)
Intra-abdominal hemorrhage	3 (4)
Vascular pathologies (aortic dissection, superior mesenteric artery stenosis)	2 (3)
Mesenteric adenitis	1 (1)
Hepatic abscess	1 (1)
Appendicitis	1 (1)
Ureteral calculus	1 (1)

*N* number

For each of the nine analyzed parameters, the available data from our study group in numbers (n) and percentage (%) are as follows: shock index, *n* = 95 (87%); fever, *n* = 92 (84%); pain intensity, *n* = 58 (53%); pain duration, *n* = 103 (94%); abnormal bowel sounds, *n* = 81 (74%); peritonism, *n* = 105 (96%); leukocytosis, *n* = 109 (100%); relative lymphopenia, *n* = 106 (97%); and CRP, *n* = 107 (98%). Only non-missing data was analyzed.

### Comparison between clinical and laboratory findings and CT results (reference standard)

Table 2 shows the demographic, clinical and biological characteristics of our study population.

**Table 2:** Baseline characteristics of patients with and without significant findings on emergency abdominal computed tomography

Characteristic	Positive CT *N* = 71	Negative CT *N* = 38	*P*-value
Mean age, years	57	62	0.2
Sex (male), N (%)	33/71 (46)	16/38 (42)	0.4
Positive shock index, N (%) Missing	5/62^a^ (8) 9	2/33^a^ (6) 5	1
Fever ≥38 °C, N (%) Missing	11/59^a^ (18) 12	4 /33^a^ (12) 5	0.56
Abdominal guarding, N (%) Missing	24/68^a^ (35) 3	10/37^a^ (27) 1	0.51
Pain duration > 6 h, N (%) Missing	55/68^a^ (81) 3	30/35^a^ (86) 3	0.6
Pain intensity ≥7/10, N (%) Missing	22/39^a^ (56) 32	13/19^a^ (68) 19	0.4
Abnormal bowel sounds, N (%) Missing	25/53^a^ (47) 18	19/28^a^ (68) 10	0.10
Elevated C-reactive protein, N (%)			
≥11 Missing	43/69^a^ (62) 2	22/38^a^ (58) 0	0.68
≥ 20 Missing	35/69^a^ (51) 2	18/38^a^ (47) 0	0.84
≥40 Missing	30/69^a^ (43) 2	11/38^a^ (29) 0	0.15
Leukocytosis, N (%) Missing	38/71 (54) 0	10/38 (26) 0	0.008
Relative lymphopenia, N (%) Missing	48/69^a^ (70) 2	14/37^a^ (38) 1	0.002
Association of leukocytosis & relative lymphopenia, N (%)	33/69^a^ (48)	4/37^a^ (11)	≤ 0.001
Missing	2	1	

*N* number

^a^Numerator corresponds to the number of patients who met the evaluated criteria; denominator corresponds to the total number of examinations, after removing the missing data

No difference was observed between patients with and without significant findings on CT with regards to mean age, gender, presence of positive shock index, fever, presence of abdominal guarding, pain duration, and intensity, presence of abnormal bowel sounds, or elevated CRP. CRP was analyzed using a cutoff value of 11 mg/l, as well as a threshold value of the median (20 mg/l) and the double of the median (40 mg/l). None of these CRP thresholds were associated with significant findings on CT. Leukocytosis and relative lymphopenia were associated with positive CT examinations (*p* = 0.008 and *p* = 0.002, respectively). The simultaneous presence of leukocytosis and relative lymphopenia was also associated with a positive CT (*p* < 0.001). Multivariate analysis confirmed that only the simultaneous presence of leukocytosis and relative lymphopenia was significantly associated with positive CT findings (Table 3).

**Table 3:** Logistic regression modeling of positive computed tomography findings as a function of leukocytosis, relative lymphopenia, and an association of both parameters

	Multivariate analysis	
	Odds ratio (95% CI)	*P* value
Leukocytosis	0.67 (0.15–2.8)	0.58
Relative lymphopenia	1.5 (0.52–4.2)	0.45
Association leukocytosis & relative lymphopenia	8.25 (1.19–57.5)	0.033

*CI* confidence interval

For leukocytosis, the sensitivity for predicting a positive CT was 53.5% (38/71), specificity 73.7% (28/38), positive predictive value (PPV) 79% (38/48), and negative predictive value (NPV) 45.9% (28/61). For relative lymphopenia, these values were, respectively, 69.6% (48/69), 62.2% (23/37), 77.4% (48/62) and 47.7% (21/44). The simultaneous presence of leukocytosis and relative lymphopenia has a high specificity and PPV, both 89.2% (33/37), for predicting a significant pathology on CT. The sensitivity and NPV, however, are limited, with both 47.8% (33/69) (Table 4).

**Table 4:** Sensitivity, specificity, PPV, and NPV for predicting a significant diagnosis on abdominal computed tomography

	Sensitivity	Specificity	PPV	NPV
Leukocytosis	53.5 (38/71)	73.7 (28/38)	79.2 (38/48)	45.9 (28/61)
Relative lymphopenia	69.6 (48/69)	62.2 (23/37)	77.4 (48/62)	47.7 (21/44)
Association of leukocytosis & relative lymphopenia	47.8 (33/69)	89.2 (33/37)	89.2 (33/37)	47.8 (33/69)

*PPV* positive predictive value, *NPV* negative predictive value

## Discussion

The aim of our study was to determine which of the clinical and laboratory variables obtained at patients’ admission to an emergency department were predictive of the presence of significant pathology on CT. Our results show that the only variables meaningfully associated with the presence of a significant pathology on CT are leukocytosis and relative lymphopenia, and that the joint presence of these two anomalies may warrant an abdominal CT on its own merit. However, none of the parameters could be used to rule out a significant pathology.

Some studies have suggested that leukocytosis is associated with the presence of pathology on the CT of patients presenting for acute abdominal pain. Thus, in an Australian retrospective study conducted in 2006 by L. Modahl et al., the authors showed that the presence of leukocytosis was a good predictor of a positive CT, as well as the pediatric age and the presence of specified pre-CT diagnosis [19]. Leukocytosis was the only parameter analyzed in both this study and in ours. However, as no pediatric cases were included in our study, our results cannot be accurately compared to this latter study. A recent study indicated that leukocytosis, together with CRP, is a good predictor of a positive CT [20], particularly in patients with right iliac fossa pain. Our study only partially confirms these results as it does effectively show an association between leukocytosis and the presence of abdominal pathology on CT, but it does not confirm the predictive role of CRP and patients with right iliac fossa pain were not included.

An association between relative lymphopenia and appendicitis has been reported in scientific literature [21, 22], but, to our knowledge, there have been no studies to directly correlate relative lymphopenia with CT results in patients presenting with abdominal pain where appendicitis was not suspected. Lymphopenia has, however, been reported as a marker of stress [23] and of infectious pathologies [24]. More broadly, it has been identified as a good predictor of bacteremia in emergency departments [25], and reported as better than CRP or leukocyte count [26]. Its presence has also been noted as a negative prognosis factor in surgical intensive care unit patients [24]. Based on these studies, it is not surprising that our analysis revealed an association between lymphopenia and significant pathology on CT.

CRP, on the other hand, was of no interest in the prediction of CT pathology, regardless of the threshold used in the frame of this study. Our results support those of a prospective study which noted no significant difference in the CRP values in patients with non-specific and non-severe abdominal pain compared with patients with severe abdominal pain [27]. Furthermore, CRP may stay low despite serious acute conditions if tested very early or if the patient has taken drugs with anti-inflammatory effects, such as non-steroidal anti-inflammatory drugs, corticosteroids, or antibiotics, before consultation [28, 29].

A meta-analysis has nonetheless shown that the combination of a CRP threshold of > 50 and elevated leukocytosis (> 15 G/L) had a high PPV for differentiating between emergency abdominal pathologies, requiring CT, and those which are not emergencies. However, only 8.7% of patients in this study presented with these two variables [29], meaning that this criteria is not useful in a routine clinical setting.

The clinical variables analyzed were not predictive of the presence of significant pathology on CT. Several studies have also demonstrated the overall limitations of clinical variables in the evaluation of acute abdominal pain, with specificity often reported as less than 50% [12] and accuracy between 47 and 76% [13–16]. However, it has been established that the value of clinical examinations varied between abdominal conditions, with clinical examinations reported as more effective with abdominal conditions in which pain is localized [7–9, 11, 20], compared with widespread pain. Our study population did not include these suspected diagnoses, apart from diverticulitis, for which a care plan is usually put in place based on clinical examination. The patients in our study presented with less specific abdominal pain with a variety of causes, which explains the difficulty in finding common clinical signs among the different conditions found, and, by extension, the weakness of the clinical variables in the prediction of significant pathology on CT.

Similarly, some conditions found on CT, such as bowel obstruction, often do not cause fever. Others, such as diverticulitis, are not associated with abnormal bowel sounds. Furthermore, fever may not be present in older patients, or those who have taken antipyretic drugs. Pain with a duration of over 6 h before admission was also not relevant according to our analysis. This concurs with Eskelinen M et al.’s observations in a broad prospective study of 1333 cases, which evaluated, among others, how well pain duration predicted severity [30].

Combining leukocytosis and relative lymphopenia, we obtained a specificity of nearly 90% for the prediction of significant pathology on CT. If CT was routinely ordered in the presence of this combination of signs, more than one third (34%, 37/106) of the patients in our study could have benefited from accelerated care, without the need for other preliminary investigations or in-depth clinical examinations. Such a process has potential to improve patient flow, reduce morbidity and save medical resources.

On the other hand, due to a low sensitivity  and negative predictive value (48%), the absence of leukocytosis and lymphopenia does not indicate a lack of significant pathology on CT. The selection of patients with non-specific abdominal pain who do not require CT must continue to be based on the assessment by a clinician, by means of a series of clinical and laboratory tests [11, 30].

Our study has several limitations. First, it was a pilot study, focusing on a limited number of patients at a single hospital. It is also possible that variables which were not significant in our study could in fact be significant among a larger cohort of patients and could form the basis of a predictive score. However, the main aim of our study was to identify the variables which best predicted the presence of a serious pathology, which were already significant in a small group. In addition, due to its retrospective design, we were confronted with a certain amount of missing data, particularly with regards to patients’ medical history and clinical examination.

Furthermore, our study population was subject to a possible selection bias, as it was made up of patients who had already been selected for CT scanning, that is, patients whose clinicians suspected significant abdominal pathology based on the examinations already undertaken. The number of patients with abdominal pain who were not referred for CT scanning, however, is not known; it is thus not possible to determine whether the criteria we have deemed predictive of significant pathology on CT, namely leukocytosis and relative lymphopenia, would still be significant without prior triage by a clinician. A prospective study on all patients presenting with abdominal pain, with systematic examination of these laboratory tests is therefore recommended to confirm the results of this pilot study.

## Conclusion

In conclusion, the results of our preliminary study suggest that the association of leukocytosis and relative lymphopenia may predict the presence of significant pathology on CT, with a specificity and PPV close to 90%. We recommend the undertaking of prospective studies to confirm these results, in which case the decision to order a CT scan could be accelerated in more than 30% of patients presenting with abdominal pain, not suspected of appendicitis or renal colic.

## Abbreviations

CRP: C-reactive protein
CT: Computed tomography
IQR: Inter-quartile range
NPV: Negative predictive value
PPV: Positive predictive value
WBC: White blood cell
